# The Impact of Prior COVID-19 on Long-Term Mortality and Echocardiographic Predictors in Chronic Kidney Disease Patients

**DOI:** 10.3390/diagnostics15060678

**Published:** 2025-03-10

**Authors:** Laura Vasiliu, Gianina Dodi, Mihai Onofriescu, Anca Diaconu, Luminita Voroneanu, Radu A. Sascau, Cristian Statescu, Adrian C. Covic

**Affiliations:** 1 Faculty of Medicine, Grigore T. Popa University of Medicine and Pharmacy of Iasi, 700115 Iasi, Romania; laura.tapoi@yahoo.com (L.V.); mihai.onofriescu@gmail.com (M.O.); lumivoro@yahoo.com (L.V.); radu.sascau@gmail.com (R.A.S.); cstatescu@gmail.com (C.S.); accovic@gmail.com (A.C.C.); 2 Prof. Dr. George I.M. Georgescu Institute of Cardiovascular Diseases, 700503 Iasi, Romania; anca.a.diaconu@gmail.com; 3 Faculty of Medical Bioengineering, Grigore T. Popa University of Medicine and Pharmacy of Iasi, 700115 Iasi, Romania; 4 Academy of Romanian Scientists, 050044 Bucharest, Romania; 5 Academy of Medical Sciences, 030173 Bucharest, Romania

**Keywords:** cardiac dysfunction, chronic kidney disease, COVID-19, hemodialysis, survival

## Abstract

**Background/Objectives**: Chronic kidney disease (CKD) patients are at an increased risk for severe outcomes following a COVID-19 infection. However, the long-term impact of prior COVID-19 on survival in this population remains unclear. This study evaluates the association between a prior COVID-19 infection, echocardiographic parameters, and mortality in CKD patients over a 24-month follow-up period. **Methods**: A prospective cohort study was conducted on 239 CKD patients, including those on hemodialysis. A Kaplan–Meier survival analysis and Cox proportional hazards models were used to assess the impact of COVID-19, age, and comorbidities on the mortality rate. A mediation analysis examined the role of age and the echocardiographic parameters in this relationship. **Results**: Kaplan–Meier curves demonstrated a significantly lower 24-month survival probability in the COVID-19 group compared to controls (72.5% vs. 87.1%, *p* = 0.01), particularly in hemodialysis patients (62.5% vs. 83.8%, *p* = 0.01). In contrast, no significant survival difference was observed in the non-dialysis CKD patients (*p* = 0.52). A multivariate Cox analysis confirmed age as the key mediator, with COVID-19 losing significance after the age adjustment (*p* = 0.05). An echocardiographic analysis identified left and right ventricular dysfunction as independent mortality predictors, with right ventricular dysfunction partially mediating the COVID-19–mortality relationship. **Conclusions**: COVID-19 significantly reduces the survival rate in CKD patients, while left and right dysfunction are strong predictors of mortality. Age partially mediates the relationship between COVID-19 and mortality, but COVID-19 remains an independent risk factor. These findings underscore the need for cardiac function monitoring in post-COVID-19 CKD patients to improve long-term outcomes.

## 1. Introduction

The prevalence of chronic kidney disease (CKD) and its progression to end-stage renal disease is intensifying worldwide, with projections estimating that by 2040 CKD will be the 5th leading cause of death [1]. Worldwide over 2 million people are living with kidney failure, with the kidney replacement therapy (KRT)–dialysis or kidney transplantation [2] treatments as the only options, but it is possible that this number may simply represent 10% of people with CKD who currently receive treatment. It is estimated that by 2030, the number of dialysis patients will reach 5.5 million [3]. Despite the advances in treatment, mortality among dialysis patients remains high, with cardiovascular (CV) diseases accounting for nearly half of all deaths in this population [4].

COVID-19, caused by the SARS-CoV-2 virus, has led to over 700 million infections and 7 million deaths globally [5], disproportionately affecting individuals with pre-existing conditions such as CKD, diabetes, and CV disease. In dialysis patients, the combined burden of immunosuppression, chronic inflammation, and cardiac dysfunction may worsen COVID-19 outcomes, but long-term survival data remain limited [6,7].

From 2020 up until now, various observational studies evaluated post-COVID-19 effects on kidneys, and the evidence suggests modifications in all outcomes, from increased mortality, major adverse cardiovascular events (MACE), major adverse kidney events (MAKE), substantial decline in estimated glomerular filtration rate (eGFR) within 6/12 or 24 months from the infection date, new-onset CKD, and so on, as detailed in Table 1.

The review paper by Long et al. [14] examines the long-term effects (CKD incidence and progression) in COVID-19 patients. After analyzing the available systematic reviews, meta-analyses, and 24 original articles reporting AKI recovery rates in patients with severe COVID-19, it was summarized that both AKI and CKD are associated with severe COVID-19 and the risk of death. COVID-19-induced AKI may lead to CKD via tubular, endothelial, and glomerular injuries that occur in patients with severe disease.

It is clear that long COVID-19 puts patients at a high risk of CKD and close monitoring of kidney and heart functions is necessary among the post-COVID-19 patients.

Echocardiography is a key tool for assessing CV risk in high-risk populations, and studies in the general population have shown significant echocardiographic changes in COVID-19 patients. Acute phase studies report right ventricular (RV) dysfunction, which is strongly linked to mortality, in up to 40% of COVID-19 patients [15,16]. Additionally, LV global longitudinal strain (GLS) impairment has been associated with increased in-hospital mortality in SARS-CoV-2 patients [17,18]. However, the long-term impact of prior COVID-19 on cardiac function and survival, particularly in CKD and hemodialysis (HD) patients, is not well understood.

Given these gaps, this prospective cohort study aims to evaluate the impact of prior COVID-19 infection on 24-month mortality in CKD patients, with a particular focus on cardiac function, as assessed by an echocardiography. We hypothesize that prior COVID-19 is associated with increased long-term mortality, particularly in HD patients, and that cardiac dysfunction plays a key role in mediating this effect. Additionally, we investigate the role of age as a potential mediator, given its established influence on CKD progression and CV mortality.

## 2. Materials and Methods

### 2.1. Study Design

CARDIO SCARS IN CKDis a single-center prospective cohort study conducted from October 2021 to October 2023. This study was designed to evaluate CV risk in patients with CKD (stages 3 to 5), including those undergoing HD, and kidney transplant (KTx) recipients with a prior SARS-CoV-2 infection. All participants were required to have fully recovered from the acute phase of the SARS-CoV-2 infection. These patients were compared to a control group consisting of patients with similar CKD stages but without prior COVID-19. The study is listed in the ClinicalTrial.gov database under the identifier NCT05125913 and the protocol was previously published [19] by our group.

The primary outcome of this study was all-cause mortality.

### 2.2. Setting

The study was conducted at Dr. C.I. Parhon University Hospital in Iasi, Romania, and additionally included patients from dialysis units affiliated with the hospital. Data collection took place between October 2021 and October 2022, with a follow-up period extending until October 2023. The study was conducted during the mid-phase of the COVID-19 pandemic, with Delta and Omicron as the predominant variants. The Delta variant, which was linked to higher viral loads, increased transmissibility, and greater hospitalization rates, was overtaken by the Omicron variant in late November 2021. The Omicron variant spread rapidly but caused milder infections with lower hospitalization rates. At the study’s start in September 2021, Romania’s vaccination rate was 33%, rising to 42.55% by the end of the study [20,21].

Eligible participants were identified through electronic health records and direct physician referrals. This study was approved by the Ethical Committee of Grigore T. Popa University of Medicine and Pharmacy of Iasi on 26 September 2021 with the protocol code no. 110/2021.

### 2.3. Participants

Eligible participants were adults (>18 years), diagnosed with CKD stages 3–5, including those on HD or with a KTx, with prior confirmed COVID-19 disease, minimum 2 weeks after the confirmed test, and who have fully recovered from the acute phase of SARS-CoV-2 infection. The control group included age, sex, and kidney disease matched patients without prior confirmed SARS-CoV-2 infection. Patients with a prior diagnosis of pulmonary fibrosis, pneumectomy, or massive pleural effusion, active malignancies, pregnancy, or congenital heart disease were excluded.

After confirming eligibility, informed consent was obtained from each participant, and, afterwards, the contributors attended a baseline assessment, including a detailed interview and completion of the trial datasheet. When necessary, additional data were retrieved from medical records. After that, the patients underwent an echocardiography, performed by specialized physicians. The patients continued their usual medical care and received copies of their records after each visit, which was also available to their physician if needed. Participants underwent clinical evaluations every six months for up to one year. Follow-up data were collected through in-person visits, electronic health records, and telephone interviews when necessary.

### 2.4. Variables

The primary outcome of this study was the all-cause mortality rate, defined as death from any cause during the follow-up period.

The main exposure variable was the SARS-CoV-2 infection, diagnosed based on WHO interim guidelines using the reverse transcriptase polymerase chain reaction (RT-PCR) assay performed on nasal or throat swabs [22]. Among the key predictors analyzed were echocardiographic parameters assessing the left and right ventricular (L/RV) systolic functions. Potential confounders included demographic characteristics and pre-existing comorbidities.

### 2.5. Data Measurements

At baseline, various demographic parameters were recorded, including age, sex, height, body weight, CKD etiology, dialysis/KTx vintage, smoking status, alcohol consumption history, diabetes, heart failure, coronary heart disease, chronic pulmonary disease, and chronic medication use. Additionally, COVID-19-related data were collected, such as the diagnosis date, disease severity, and the need for hospital admission, oxygen therapy, or other specific treatments.

All echocardiographic evaluations were conducted following the guidelines of the American Society of Echocardiography and the European Association of Cardiovascular Imaging [23], using the Philips CX50 ultrasound system (Andover, MA, USA) and QLAB 7.1 software (Andover, MA, USA).

All patients underwent a comprehensive echocardiographic evaluation, assessing cardiac anatomy (diameters, volumes, mass) and function, including LV and RV systolic performance, LV diastolic function, and pulmonary hypertension parameters. For this analysis, key systolic function measures included LV ejection fraction (EF) and GLS, and for RV function, fractional area change (FAC), tricuspid S’ velocity, and right ventricular free wall longitudinal strain (RVFWLS). LV EF was calculated using Simpson’s biplane method, and GLS was obtained from three standard apical views using QLAB 7.1 software. High-quality cine loops ensured accurate ROI estimation, manually adjusted for LV thickness, with GLS < −18% considered normal. RV function was assessed via FAC, tricuspid S’ (tissue Doppler), and RVFWLS from a modified apical four-chamber view using QLAB 7.1. The mean RVFWLS of the three free wall segments was recorded, with <−23% as the normal threshold. All measurements were observer-validated for accuracy.

### 2.6. Bias

Selection bias was minimized by including all consecutive eligible CKD patients from the participating centers. To minimize misclassification bias, a prior COVID-19 infection was confirmed through documented RT-PCR results and clinical history. Echocardiographic measurements were performed using standardized protocols by trained cardiologists, and intra- and inter-observer variability was assessed in a random subset of patients. To minimize variability due to volume status, echocardiography was performed on the second day after a dialysis session, during the short interdialytic interval.

Confounding was addressed using multivariable Cox proportional hazards models adjusting for age, sex, comorbidities, dialysis status, and echocardiographic parameters. Mediation analysis was conducted to explore the role of age and RV dysfunction in the COVID-19-mortality relationship. Multicollinearity was ruled out using variance inflation factors (VIF < 2).

### 2.7. Study Size

The required sample size was estimated to ensure adequate power for detecting differences in all-cause mortality, the primary outcome of this study. Sample size calculations were performed for Cox proportional hazards regression models and Chi-square tests to compare mortality rates between the groups.

For Cox regression models, using Schoenfeld’s formula, we estimated that a minimum of 160 participants was required to achieve 80% power at a significance level of 0.05. A Chi-square test was planned to compare mortality rates between COVID-19-positive and COVID-19-negative CKD patients. Based on an expected mortality rate of 22% in the COVID-19 group [24,25,26,27,28] and 9% in the control group [29], and assuming α = 0.05 and 80% power, the required total sample size was estimated to be 236 participants. For the comparison of echocardiographic predictors between groups, independent sample *t*-tests were used. Assuming a moderate effect size (Cohen’s d = 0.5), 80% power, and a two-tailed α = 0.05, the required sample size was 64 participants per group.

All calculations were performed using standard statistical formulas and validated with G*Power 3.1 to ensure robust estimation. A final sample size exceeding these minimum requirements was targeted to account for potential dropouts and missing data.

### 2.8. Quantitative Variables

Continuous variables were reported as mean ± standard deviation (SD) if normally distributed and median with interquartile range (IQR) if non-normally distributed, as assessed using the Shapiro–Wilk test. Categorical variables were expressed as absolute numbers (n) and percentages (%).

Age was analyzed as a continuous variable in Cox proportional hazards models and was further stratified into tertiles for a descriptive analysis to explore potential non-linear associations with mortality.

Echocardiographic parameters, including LV EF and GLS, tricuspid annular plane systolic excursion (TAPSE), RVFWLS were primarily analyzed as continuous variables. For subgroup analyses, LV dysfunction was defined as LVEF < 40% or GLS > −18% and RV dysfunction was defined as RVFWLS > −23%, FAC < 35% or tricuspid S’ < 9.5 cm/s, based on guideline-recommended cut-offs.

Comorbidities (hypertension, diabetes, atrial fibrillation, heart failure, ischemic heart disease, and pulmonary disease) were treated as binary variables (yes/no).

In survival analyses, time-to-event (mortality) was analyzed as a continuous variable in months, with right-censoring at 24 months for patients who remained alive at the end of the follow-up period.

### 2.9. Statistical Methods

A statistical analysis was performed using IBM SPSS Statistics 26.0. Continuous variables were compared using independent samples *t*-test, while categorical variables were compared using the Chi-square test.

The survival analysis was conducted using Kaplan–Meier curves to compare the survival between groups, with the statistical significance assessed using the log-rank test. The time variable was defined as the duration (in months) from baseline to the event of interest (death), with censoring applied to participants who remained alive at the study’s end.

To identify the independent predictors of survival, a Cox proportional hazards regression model was used, estimating hazard ratios (HR) with 95% confidence intervals (CI). The proportional hazards assumption was tested to confirm the model’s validity. No adjustments were made for potential confounders in the Kaplan–Meier analysis, but Cox regression adjusted for covariates including age, comorbidities, and echocardiographic parameters.

A mediation analysis was conducted to examine whether several factors (age, LV and RV systolic dysfunction) mediate the relationship between COVID-19 and mortality using the PROCESS macro (Model 4) for SPSS (http://afhayes.com/spss-sas-and-r-macros-and-code.html, accessed on 15 February 2025). A logistic regression-based mediation model with 5000 bootstrap resamples was employed to estimate the indirect effect of COVID-19 on mortality caused by each factor. The direct effect (c’ path) represents the relationship between COVID-19 and mortality after adjusting for the factor, while the indirect effect (a × b path) evaluates whether the factor partially or fully mediates this association. Statistical significance was determined if the bootstrapped 95% CI did not include zero.

A *p*-value < 0.05 was considered statistically significant for all analyses.

## 3. Result

### 3.1. Baseline Characteristics

**Figure 1:**
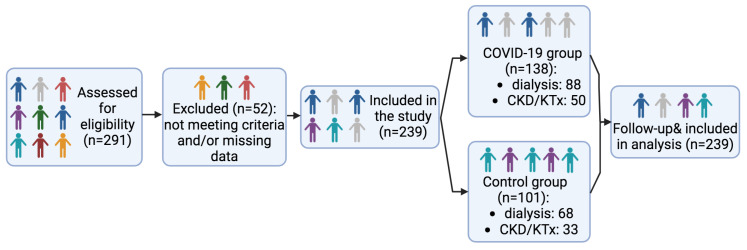
Cohort study flow diagram.

A total of 239 patients were included in this study: 138 in the COVID-19 group and 101 in the control group (Figure 1). As expected from previous reports, COVID-19 patients were significantly older (*p* = 0.02), and had a higher prevalence of diabetes (*p* = 0.002), heart failure (*p* = 0.03), and ischemic heart disease (*p* = 0.008). The average time between the COVID-19 diagnosis and the baseline evaluation was 1.06 ± 0.67 months.

LV systolic dysfunction was observed in 39.1% (54 patients) of the COVID-19 group and 33.7% (34 patients) of the control group (*p* = 0.38). RV systolic dysfunction was present in 29.7% (41 patients) of the COVID-19 group and 16.8% (17 patients) of the control group (*p* = 0.02).

The dialysis group comprised 156 patients, 88 in the COVID-19 group and 68 in the control group. The COVID-19 group had a mean dialysis vintage of 57.7 ± 62.7 months, while the control group had a mean dialysis vintage of 74.1 ± 77.3 months. The CKD group, including CKD patients and KTx patients, comprised 83 individuals, with 50 in the COVID-19 group and 33 in the control group. The mean eGFR for CKD patients was 21.01 ± 13.09 mL/min/1.73 m^2^ in the COVID-19 group and 16.24 ± 7.57 mL/min/1.73 m^2^ in the control group. For KTx patients, the mean eGFR was 56.91 ± 20.18 mL/min/1.73 m^2^ in the COVID-19 group and 55.06 ± 22.29 mL/min/1.73 m^2^ in the control group. The distribution of patients across the CKD stages is provided in Table 2.

The detailed demographics are provided in Table 3.

### 3.2. Survival Analysis

All patients were followed for two years. In the COVID-19 group, 38 deaths were recorded, while the control group reported 13 deaths. Among dialysis patients, there were 44 deaths—33 in the COVID-19 group and 11 in the control group. In the CKD group, a total of 7 deaths occurred, with 5 in the COVID-19 group and 2 in the control group.

Non-surviving CKD patients were significantly older across both groups. Aside from a higher prevalence of atrial fibrillation among COVID-19 non-survivors, there were no significant differences in baseline comorbidities between the groups. In both groups, LV GLS and RVFWLS were significantly more impaired in patients who did not survive. Furthermore, LV EF and tricuspid S’ were lower in the deceased patients, but this difference was observed only in the control group (Table 4).

### 3.3. COVID-19 as a Predictor of Mortality

**Figure 2:**
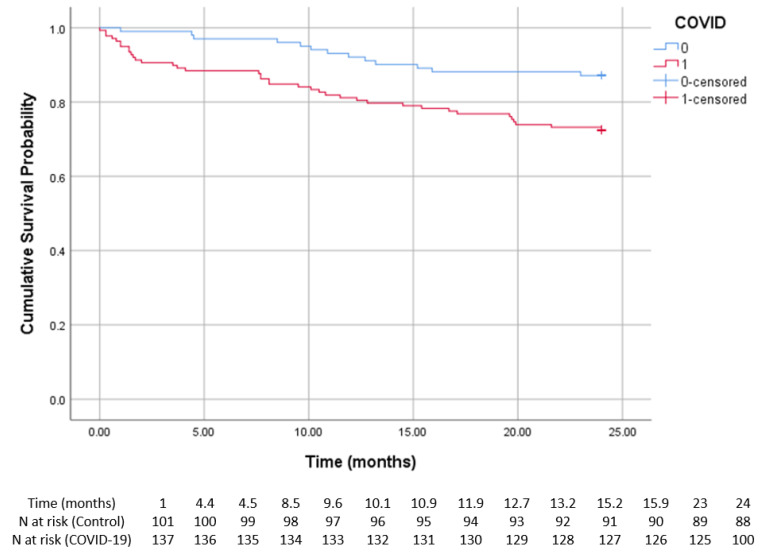
Kaplan–Meier survival curve and number of at risk patients table of all patients with and without prior COVID-19. The survival probability over time is shown for patients with (red) and without (blue) COVID-19. The vertical marks indicate censored cases. The number at risk at various time points is provided in Table 3. The COVID-19 group showed significantly lower survival rates compared to the control group (log-rank < 0.01).

The Kaplan–Meier survival curves (Figure 2) illustrate the cumulative survival probabilities over 24 months in patients with and without prior COVID-19. The prior COVID-19 group exhibited a lower survival probability throughout the study period. By 12 months, the survival probability was 88.1% in the control group and 91.3% in the COVID-19 group. However, by the end of the follow-up, survival probability declined to 87.1% in the control group and 72.5% in the COVID-19 group.

A more rapid decline in survival was observed in the COVID-19 group, reflecting increased mortality associated with infection. A log-rank test confirmed a statistically significant difference between the two survival curves (*p* < 0.01).

**Figure 3:**
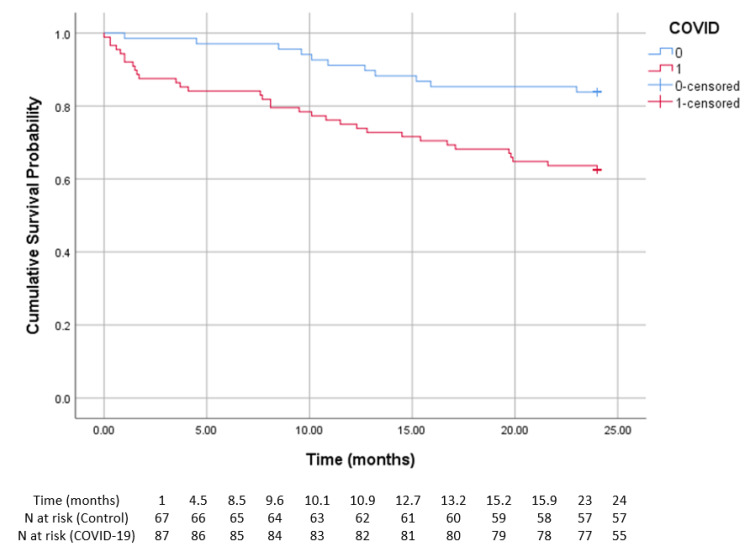
Kaplan–Meier survival curve and number of at risk hemodialysis patients with and without prior COVID-19. The survival probability over time is shown for patients with (red) and without (blue) COVID-19. The vertical marks indicate censored cases. The number at risk at various time points is provided in Table 4. The COVID-19 group showed significantly lower survival rates compared to the control group (log-rank < 0.01).

The results remained statistically significant in the dialysis group (Figure 3). At 12 months, the survival probability was 85.3% in the control group and 88.6% in patients with prior COVID-19. By 24 months, survival probability declined to 83.8% in the control group and 62.5% in the prior COVID-19 group. The number of patients at risk at various time points is provided in Table 4. A more rapid decline in survival was observed in patients with prior COVID-19, reflecting increased mortality associated with infection. A log-rank test confirmed a statistically significant difference between the two survival curves (log-rank *p* < 0.01).

**Figure 4:**
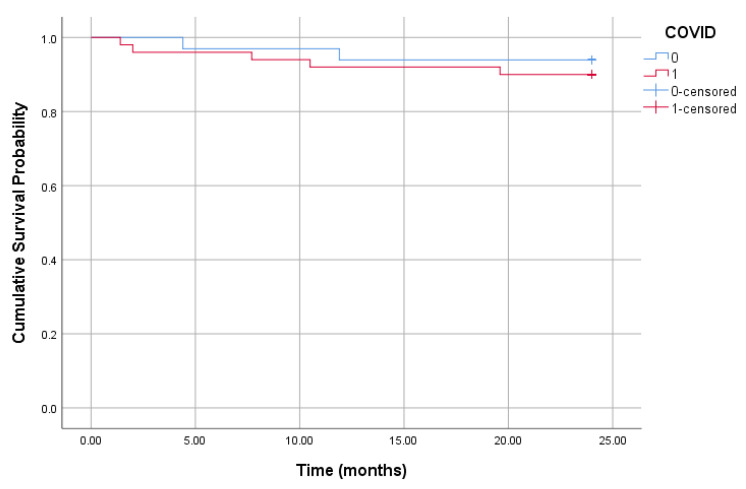
Kaplan–Meier survival curve of CKD patients with and without prior COVID-19. The survival probability over time is shown for patients with (red) and without (blue) COVID-19. The vertical marks indicate censored cases. There were no significant differences in survival rates between groups (log-rank *p* = 0.52).

In the CKD patients, there were no significant differences in survival rates between groups (log-rank *p* = 0.52)|(Figure 4).

### 3.4. Univariate Analysis and Multivariate Cox Proportional Hazards Models

In the univariate analysis conducted on all 239 patients, COVID-19 was a significant predictor of death (HR 2.37, 95% CI 1.26–4.45, *p* = 0.01).

A Cox proportional hazards model was used to assess the association between COVID-19, age, and comorbidities with survival. The model included COVID-19 status (primary exposure), age (continuous variable, potential mediator/confounder), and comorbidities (hypertension, diabetes, atrial fibrillation, heart failure, ischemic heart disease, pulmonary disease). The assumption of proportional hazards was assessed using log-minus-log survival curves and Schoenfeld residuals, confirming that model assumptions were met. VIF were all < 2, indicating no evidence of multicollinearity. The following two models were compared: a full model including COVID-19, age, and comorbidities, and a reduced model including only COVID-19 and comorbidities.

In the full model, COVID-19 was borderline significant (*p* = 0.05), while age was highly significant (*p* < 0.01). However, when age was removed from the model, the significance of COVID-19 increased (*p* = 0.02) and certain comorbidities, such as hypertension and diabetes, also became significant.

To explore whether age mediated the relationship between COVID-19 and mortality, a mediation analysis was performed. COVID-19 status was significantly associated with age (OR = 4.67, SE = 1.97, *p* = 0.01, 95% CI 0.78–8.56), indicating that individuals with COVID-19 were generally older. Age, in turn, was significantly associated with an increased risk of mortality (OR = 0.07, SE = 0.01, *p* < 0.01, 95% CI 0.04–0.10), confirming that older individuals had a higher likelihood of death. Even after adjusting for age, COVID-19 remained a significant predictor of mortality (OR = 0.78, SE = 0.37, *p* = 0.03, 95% CI 0.04–1.51). The bootstrapped indirect effect of COVID-19 on mortality through age was OR = 0.34, SE = 0.18, with a 95% CI 0.04–0.74, confirming a significant mediation effect.

When LV systolic dysfunction was added to the full model, it was highly significant (*p* = 0.01), but the effect of COVID-19 was attenuated. In the final model, both LV and RV systolic dysfunction were significantly associated with increased mortality (*p* = 0.01 and 0.02, respectively), and COVID-19 lost its significance.

A second mediation analysis was conducted to examine whether RV systolic dysfunction mediated the relationship between COVID-19 and mortality. COVID-19 was significantly associated with RV systolic dysfunction (OR = 2.08, SE = 0.32, *p* = 0.02, 95% CI 1.10–3.94) and RV systolic dysfunction was a strong predictor of mortality (OR = 2.56, SE = 0.34, *p* < 0.01, 95% CI 1.30–5.03). Even after adjusting after RV systolic dysfunction, COVID-19 remained a significant predictor of death (OR = 2.30, SE = 0.35, *p* = 0.02, 95% CI 1.13–4.65). The bootstrapped indirect effect of COVID-19 on mortality through RV systolic dysfunction was OR = 0.94, SE = 0.36, with a 95% CI 0.26–1.76, confirming a significant mediation effect.

In contrast, LV systolic dysfunction did not mediate the relationship between COVID-19 and mortality (OR = 0.78, se 0.27, *p* = 0.38, 95% CI 0.46–1.34).

To assess whether LV systolic dysfunction modified the relationship between COVID-19 and mortality, an interaction term was tested in a multivariate Cox proportional hazards model. The interaction between COVID-19 and LV dysfunction was not statistically significant (HR = 1.94, 95% CI: 0.51–7.38, *p* = 0.33), indicating no formal evidence of effect modification. However, an exploratory stratified analysis suggested a potential difference in the effect of COVID-19 on mortality. Among the patients without LV systolic dysfunction, COVID-19 was associated with a higher risk of mortality (HR = 2.61, 95% CI 0.92–7.39, *p* = 0.07), whereas this association was weaker in those with LV systolic dysfunction (HR = 1.45, 95% CI 0.60–3.54, *p* = 0.40) (Table 5).

### 3.5. Subgroup Analysis on Dialysis Patients

When the Cox analysis was conducted exclusively on dialysis patients, COVID-19 had a stronger and more consistent association with the outcome. Age remained a significant predictor across all models. Additionally, LV systolic dysfunction remained a strong and significant predictor, but RV systolic dysfunction lost its significance (Table 6).

## 4. Discussion

This is among the first long-term follow-up study in CKD patients demonstrating that a prior COVID-19 infection is significantly associated with a decreased survival probability over a 24 month period compared to those without a prior infection. This effect is more pronounced in HD patients, aligning with previous research demonstrating that HD patients are at heightened risk for severe COVID-19 outcomes, including increased mortality rates. There are several studies that reported the mortality rate over time post-COVID-19, such as 36% within 1 year of follow-up in HD patients [28], 13% in COVID-19 and 32% in those with AKI [8] over 90 days follow-up, 3.1% in CKD and COVID-19 patients from Bronx [10], 28% hospital mortality and 29% 90-day mortality rate on COVID-19 patients from intensive care unit in London, United Kingdom [11], and 34% over a median follow-up of 1.5 years [30].

Our study yielded similar results. The overall mortality rate was 23%, with the prior COVID-19 group experiencing a mortality rate of 27.5%, compared to 12.8% in the control group. Among dialysis patients, the mortality rate was 37.5% in patients with prior COVID-19, while it was 16.1% in the control group. Unlike previous studies that reported mortality rates over shorter periods, we assessed and reported mortality rates over a 2-year follow-up period.

In the univariate analysis, prior COVID-19 was a significant predictor of mortality in CKD; however, its effect diminished when adjusting for age and comorbidities. This suggests that age may act as both a mediator and a confounder in the relationship between COVID-19 and mortality. Mediation analysis confirmed that age partially mediates this relationship, but COVID-19 remains an independent risk factor after adjustment suggesting that while older age is a primary risk factor for mortality, the influence of comorbidities may be more pronounced in younger populations.

Further analysis revealed that LV and RV systolic dysfunction are strong independent predictors of mortality. Notably, RV dysfunction partially mediates the effect of COVID-19 on mortality, whereas LV dysfunction does not. However, an exploratory stratified analysis suggests a greater impact of COVID-19 on mortality in patients without pre-existing LV dysfunction. These findings are consistent with studies reporting that cardiac dysfunction is associated with adverse outcomes in COVID-19 patients, but on significantly shorter follow-up periods. Both LV and RV dysfunction were significant predictors of disease severity and mortality [17,31]. A LV GLS threshold of −16.5% was a strong mortality predictor [32], while a RVFWLS cut-off of −23% showed the highest sensitivity for predicting mortality in COVID-19 patients [18,33]. To the best of our knowledge, our study is the first to establish significant correlations between echocardiographic parameters and mortality rates in a CKD population with prior COVID-19 over a 24-month follow-up period.

Our study has several limitations. This study spans a period with multiple SARS-CoV-2 variants, but we could not account for their differing effects on mortality. Additionally, the lack of vaccination data is a limitation, as vaccination significantly impacts long-term outcomes in CKD patients. Selection bias may be present; underlying differences between groups might not be fully addressed, leading to potential bias. Furthermore, this is a single-center study, limiting generalizability due to potential differences in patient characteristics and treatment protocols. The relatively small sample size may also affect statistical power, particularly in subgroup analyses and adjustments for confounders. In addition, patients with prior COVID-19 did not have a previous echocardiographic evaluation, in order to truly assess the impact of COVID-19 on cardiac function. As an observational study, the results should be interpreted cautiously, as they establish associations rather than causality.

## 5. Conclusions

In conclusion, we were the first to demonstrate that left and right ventricular dysfunction plays an important predictive role in long-term mortality among CKD patients with prior COVID-19. COVID-19 significantly reduces survival in HD patients but does not significantly affect CKD patients who are not on dialysis. Age partially mediates the relationship between COVID-19 and mortality, but COVID-19 remains an independent risk factor even after adjustment. LV and RV systolic dysfunctions are strong independent predictors of mortality, with RV dysfunction partially mediating the effect of COVID-19 on mortality. LV systolic dysfunction does not significantly modify the effect of COVID-19 on mortality, but COVID-19 appears to contribute more to mortality in patients without a pre-existing LV dysfunction. These findings highlight the importance of cardiac function assessments in COVID-19 patients with CKD and HD and suggest that patients without an LV dysfunction may be at greater risk of COVID-19-associated mortality.

## Figures and Tables

**Table 1:** Outcomes in long COVID-19 according to available data.

Type of Patients	Number of Patients	Admission Period	Follow-Up	Type of Study, Location	Primary Outcome, Result	Additional Outcomes, Results	Observations	Ref.
COVID vs. Influenza	1091 vs. 1334	March–May 2020	During hospitalization, 90 days	Retrospective cohort study, Boston Massachusetts	- the risk of acute kidney injury (AKI) during hospitalization: 23 vs. 13%	- mortality: 13 vs. 2%; - mortality in those with AKI: 32 vs. 11%; - AKI recovery: 56 vs. 70%; - eGFR decline (≥25% at 90 days): 11 vs. 11%; - new CKD at ≥90 days: 14 vs. 17%.	- AKI and mortality rates are significantly higher in patients with COVID-19 than influenza; - kidney recovery times among long-term survivors appears to be similar.	[8]
		January 2017–December 2019						
COVID-19 vs. non-COVID-19	203,476 vs. 5,808,018	1 March 2020–15 March 2021; vs. 2019 data	30 days	Cohort study, Department of Veterans Health Administration healthcare system	- risk of AKI *: 59.38 vs. 25.56	- eGFR decline *: ≥30% 100.45 vs. 70.13; ≥40% 50.66 vs. 31.09; - ESKD *: 4.65 vs. 1.43; - MAKE **: 57.88 vs. 31.6 (*)	30-day survivors of COVID-19 exhibited higher risk of AKI, eGFR decline, ESKD, and MAKE than those not infected by COVID-19	[9]
CKD with COVID-19 vs. CKD without COVID-19	834 vs. 6167	3 November 2020 to 7 January 2023	6-, 12-, and 24-month	Retrospective cohort study, Bronx	- CKD progression (from stage 3a/3b to 4/5): 10.2/9.6% vs. 9.1/4.1% at 6 months; 10.2/16.5% vs. 8.6/7.3% at 12 months; 10.7/22.3% vs. 8.8/10.8% at 24 months.	- mortality: 3.1 vs. 2.3%; - MAKE ***: 2.94 at 6 months; 2.24 at 12 months; 2.03 at 24 months; - MACE *^v^: 1.73 at 6 months; 1.77 at 12 months; 1.31 at 24 months;	COVID-19 increases the risk of long-term CKD progression and cardiovascular events in patients with CKD	[10]
COVID-19	313	1 March–31 July 2020	90 days after admission	Retrospective observational study, intensive care unit in London, UK	- AKI risk within 14 days: 76.7%	- KRT: 32% (median time 3 days); - hospital mortality: 28%; - 90-day mortality: 29%; - dialysis (at discharge and 90 days): 4% vs. 5%; - renal recovery at discharge and 90 days: 81.6 vs. 90.9%; - MAKE at 90 days: 42.4%	High incidence of AKI and AKI progression in critically ill COVID-19 patients; high 90-day dialysis independence.	[11]
COVID-19 without history of kidney transplant or dialysis	2212 long COVID-19 patients	July 2020 to April 2022	12 months post-COVID-19	observational retrospective cohort study, British Columbia, Canada	- eGFR change at 1 year: 3.4% (6.7% hospitalized and 6.2% diabetes); - urine albumin–creatinine ratio (UACR): 36%.	- CKD risk: 42.4% moderate to high;	People with long- COVID experienced substantial decline in eGFR within 1 year from the infection date.	[12]
COVID-19 with AKI	3296	11 March 2020–17 August 2022	12 months post COVID-19/90 days	Single center NYC health system	- recovery at 1 year: 41.4% early recovery; 41.6% delayed recovery; 24.8% prolonged recovery;	- MACE: 17.2%; - MAKE ^v^: 22.6%; - rehospitalization: 34.7%; - recurrent AKI: 18.3%; - new-onset CKD: 23.7%, at 90 days post-COVID-19.	COVID-19 patients who develop hospital AKI are at a high risk for adverse cardiovascular and kidney outcomes, especially those with longer AKI recovery time.	[13]

* calculated as incident rate per 1000 person-years; ** defined as eGFR decline ≥ 50%, end-stage kidney disease (ESKD), or all-cause mortality; *** defined as composite of eGFR decline of  ≥30% from baseline or progression to ESKD, and presented as adjusted hazard ratio of hospitalized COVID-19 compared to non-COVID-19 patients; *^v^ defined as composite of all-cause mortality, nonfatal stroke, nonfatal myocardial infarction, and non-fatal heart failure using ICD-10 codes; ^v^ defined as a composite of dialysis dependence, death, or eGFR decline ≥ 25% from baseline at follow-up.

**Table 2:** Chronic kidney disease stages at baseline.

**CKD Group**		
**CKD Stage**	**COVID-19 Group** **N = 20**	**Control Group** **N = 6**
3a (N/%)	2 (10%)	1 (16.6)
3b (N/%)	1 (5%)	2 (33.3)
4 (N/%)	7(35%)	2 (33.3)
5 (N/%)	10 (50%)	1 (16.6)
**KTx group**		
**CKD stage**	**COVID-19 group** **N = 30**	**Control group** **N = 27**
1 (N/%)	2 (6.7)	3 (11.1)
2 (N/%)	9 (30)	5 (18.5)
3a (N/%)	9 (30)	12 (44.4)
3b (N/%)	8 (26.7)	4 (14.8)
4 (N/%)	1 (3.3)	2 (7.4)
5 (N/%)	1 (3.3)	1 (3.7)

**Table 3:** Demographics of the study population.

			CKD Etiology									
All Patients (N = 239)	Age (Mean ± SD)	Male N (%)	DM N (%)	HTN N (%)	Chronic GN N (%)	Other N (%)	HTN N (%)	DM N (%)	Afib N (%)	HF N (%)	CAD N (%)	Pulmonary Disease N (%)
COVID-19 (N = 138)	58.51 ± 14.83	75 (54.3)	23 (16.7)	15 (10.9)	32 (23.2)	68 (49.3)	112 (81.2)	44 (31.9)	27 (19.6)	65 (47.1)	43 (31.2)	8 (5.8)
Control (N = 101)	53.83 ± 15.41	56 (55.4)	12 (11.9)	10 (9.9)	31 (30.7)	48 (47.5)	78 (77.2)	15 (14.9)	12 (11.9)	29 (28.7)	17 (16.8)	5 (5)
	*p* = 0.01	*p* = 0.86					*p* = 0.45	*p* < 0.01	*p* = 0.11	*p* < 0.01	*p* = 0.01	*p* = 0.77
**HD (N = 156)**												
COVID-19 (N = 88)	62.18 ± 14.25	46 (52.3)	18 (20.5)	13 (14.8)	19 (21.6)	38 (43.2)	72 (81.8)	33 (37.5)	23 (26.1)	49 (55.7)	34 (38.6)	7 (8.0)
Control (N = 68)	57.79 ± 14.59	36 (52.9)	10 (14.7)	8 (11.8)	18 (26.5)	32 (47.1)	54 (79.4)	10 (14.7)	12 (17.6)	24 (35.3)	14 (20.6)	3 (4.4)
	*p* = 0.06	*p* = 0.93					*p* = 0.70	*p* < 0.01	*p* = 0.20	*p* = 0.01	*p* = 0.01	*p* = 0.37
**CKD (N = 83)**												
COVID-19 (N = 50)	52.04 ± 13.70	29 (58.0)	5 (10.0)	2 (4.0)	13 (26.0)	30 (60.0)	40 (80.0)	11 (22.0)	4 (8.0)	16 (32.0)	9 (18.0)	1 (2.0)
Control (N = 33)	52.04 ± 13.70	20 (60.6)	2 (6.1)	2 (6.1)	13 (39.4)	16 (48.5)	24 (72.7)	5 (15.2)	0 (0.0)	5 (15.2)	3 (9.1)	2 (6.1)
	*p* = 0.04	*p* = 0.81					*p* = 0.44	*p* = 0.43	*p* = 0.09	*p* = 0.08	*p* = 0.25	*p* = 0.33

**Table 4:** Comparison between the characteristics of survivors and deceased patients.

Variable	COVID-19 Survivors (N = 100)	COVID-19 Non-Survivors (N= 38)	*p*-Value (COVID-19)	Control Survivors (N = 88)	Control Non-Survivors (N = 13)	*p*-Value (Control)
Age (mean ± SD)	55.15 ± 14.48	67.34 ± 11.94	<0.01	51.97 ± 15.13	66.46 ± 11.01	<0.01
HTN (no., %)	83 (74.1)	29 (25.9)	0.37	88 (87.1)	13 (12.9)	0.14
Diabetes mellitus (no., %)	30 (68.2)	14 (31.8)	0.44	10 (66.7)	5 (33.3)	0.01
Ischemic Heart disease (no., %)	30 (69.8)	13 (30.2)	0.63	14 (82.4)	3 (17.6)	0.51
Atrial fibrillation (no., %)	15 (55.6)	12 (44.4)	0.02	10 (83.3)	2 (16.7)	0.67
Heart failure (no., %)	45 (69.2)	20 (30.8)	0.42	24 (82.8)	5 (17.2)	0.40
Ejection fraction (Mean ± SD) (%)	55.16 ± 10.16	54.28 ± 10.66	0.66	58.12 ± 8.48	53.00 ± 9.89	0.04
LV GLS (Mean ± SD) (%)	−17.47 ± 3.90	−15.17 ± 4.93	0.02	−18.13 ± 2.59	−14.75 ± 2.12	<0.01
FAC (Mean ± SD) (%)	41.78 ± 10.78	39.03 ± 10.89	0.18	45.72 ± 8.29	43.53 ± 8.06	0.37
Tricuspid S’ (Mean ± SD) (cm/s)	13.17 ± 2.75	12.18 ± 2.84	0.06	13.23 ± 2.67	11.20 ± 3.87	0.01
RVFWLS (%)	−18.91 ± 4.40	−16.16 ± 6.57	0.03	−20.70 ± 2.80	−18.64 ± 2.80	0.04

**Table 5:** Multivariate Cox proportional hazard models on all patients.

Univariate Analysis			
	HR	95% CI	*p*
COVID-19	2.37	1.26–4.45	<0.01
**Multivariate analysis-full model**			
COVID-19	1.87	0.97–3.57	0.05
AGE	1.05	1.03–1.08	<0.01
HTN	0.67	0.34–1.30	0.23
AFib	1.21	0.59–2.45	0.59
HF	0.93	0.42–2.04	0.86
Ischemic HD	0.95	0.43–2.10	0.90
DM	1.34	0.70–2.56	0.37
Pulmonary disease	0.95	0.33–2.72	0.93
**Multivariate analysis-reduced model**			
COVID-19	2.06	1.08–3.95	0.02
HTN	0.52	0.27–1.00	0.05
Afib	1.85	0.92–3.72	0.08
HF	0.98	0.45–2.13	0.97
Ischemic HD	1.04	0.48–2.27	0.90
DM	1.92	1.02–3.61	0.04
Pulmonary disease	1.15	0.40–3.29	0.78
**Multivariate analysis-model 3**			
COVID-19	1.77	0.92–3.39	0.08
AGE	1.06	1.03–1.09	<0.01
HTN	0.79	0.41–1.54	0.50
Afib	1.19	0.59–2.39	0.61
HF	0.87	0.39–1.93	0.74
Ischemic HD	0.70	0.30–1.61	0.40
DM	1.53	0.80–2.94	0.19
Pulmonary disease	1.08	0.38–3.07	0.87
LV systolic dysfunction	2.53	1.38–4.62	0.01
**Multivariate analysis-model 4**			
COVID-19	1.73	0.90–3.30	0.09
AGE	1.06	1.03–1.08	<0.01
HTN	0.74	0.38–1.45	0.39
Afib	1.18	0.59–2.39	0.62
HF	0.78	0.35–1.75	0.55
Ischemic HD	0.69	0.30–1.61	0.40
DM	1.52	0.78–2.94	0.21
Pulmonary disease	1.09	0.38–3.11	0.85
LV systolic dysfunction	2.27	1.23–4.18	0.01
RV systolic dysfunction	1.94	1.08–3.50	0.02

**Table 6:** Multivariate Cox proportional hazard models on dialysis subgroup.

Univariate Analysis			
	HR	95% CI	*p*
COVID-19	2.71	1.37–5.37	<0.01
**Multivariate analysis-full model**			
COVID-19	2.15	1.06–4.36	0.03
AGE	1.03	1.01–1.06	<0.01
HTN	0.57	0.26–1.25	0.16
AFib	1.16	0.57–2.37	0.66
HF	1.11	0.50–2.43	0.79
Ischemic HD	0.82	0.36–1.86	0.64
DM	1.87	0.93–3.76	0.07
Pulmonary disease	0.85	0.28–2.54	0.77
**Multivariate analysis-reduced model**			
COVID-19	2.25	1.10–4.59	0.02
HTN	0.49	0.22–1.05	0.06
Afib	1.42	0.70–2.88	0.32
HF	1.07	0.49–2.31	0.85
Ischemic HD	0.84	0.38–1.88	0.68
DM	2.37	1.19–4.70	0.01
Pulmonary disease	0.98	0.32–2.92	0.97
**Multivariate analysis-model 3**			
COVID-19	2.09	1.03–4.26	0.04
AGE	1.04	1.01–1.07	<0.01
HTN	0.64	0.29–1.40	0.26
Afib	1.18	0.58–2.39	0.63
HF	1.05	0.47–2.33	0.89
Ischemic HD	0.60	0.25–1.43	0.25
DM	2.18	1.09–4.36	0.02
Pulmonary disease	1.05	0.35–3.12	0.92
LV systolic dysfunction	2.82	1.47–5.40	<0.01
**Multivariate analysis-model 4**			
COVID-19	2.07	1.02–4.20	0.04
AGE	1.04	1.01–1.07	<0.01
HTN	0.64	0.29–1.40	0.26
Afib	1.22	0.60–2.48	0.58
HF	0.95	0.42–2.15	0.91
Ischemic HD	0.60	0.25–1.45	0.26
DM	2.21	1.09–4.48	0.02
Pulmonary disease	1.06	0.35–3.17	0.91
LV systolic dysfunction	2.45	1.25–4.78	<0.01
RV systolic dysfunction	1.74	0.92–3.29	0.08

## Data Availability

The research data are available upon request at the correspondent author.

## Abbreviations

The following abbreviations are used in this manuscript:

Afib	Atrial fibrillation
AKI	Acute kidney injury
CAD	Coronary artery disease
CI	Confidence intervals
CKD	Chronic kidney disease
CV	Cardiovascular
DM	Diabetes mellitus
eGFR	Estimated glomerular filtration rate
EF	Ejection fraction
ESKD	End-stage kidney disease
FAC	Fractional area change
FWLS	Free wall longitudinal strain
GLS	Global longitudinal strain
GN	Glomerulopathy
HD	Hemodialysis
HTN	Hypertension
HF	Heart failure
HR	Hazard ratios
IQR	Interquartile range
KRT	Kidney replacement therapy
KTx	Kidney transplant
LV	Left ventricular
MACE	Major adverse cardiovascular events
MAKE	Major adverse kidney events
n	Absolute numbers
no.	Number
RV	Right ventricular
RT-PCR	Reverse transcriptase polymerase chain reaction
TAPSE	Tricuspid annular plane systolic excursion
SD	Standard deviation
VIF	Variance inflation factors
